# Who Are We to Believe?

**Published:** 1894-10-27

**Authors:** 


### Editor's Letter-Box.

[Our correspondents are reminded that proxility is a great bar to publication, and that brevity of style and conciseness of statement greatly facilitate early insertion.]

### WHO ARE WE TO BELIEVE?

"C. L." writes: You print, "Bad air is by no means so speedy in making its effect felt as bad water . . . The death rate is an infallible danger signal." Some time ago I had to give evidence at Evesham respecting the salubrity of the natural water supply of the village, Cleeve Prior, with a view of protecting the defenceless villagers against an enforced artificial supply to be imported on payment, and against the will of the inhabitants. I took exactly your grounds, as opposed to what Mr. Balfour called "the despotism of the laboratory," and which Lord Salisbury termed an Oxford opinion, "purely hypothetical." The official officer of health disagreed with me. Here is the report:

Mr. Lunn asked if the condemnation was based upon evidence or hypothesis.

Mr. Fosbroke said the analyst had answered that.

Mr. Lunn said science was in a regular confusion and chaos. If health and strength and long life be not evidence, he was done.

Mr. Fosbroke: Absolutely no evidence.

Mr. Lunn: And where people die and get thin, miserable, and short-lived, would that be evidence of ——?

Mr. Fosbroke: None, whatever.

Mr. Lunn: Then it is pure hypothesis?

Mr. Fosbroke: Nothing of the sort,

—*Evesham Journal*, August 4th, 1894.

Of course, I was blustered down, and the peaceful, weak inspector convinced, so the Local Government Board nonsuited me. But either you are right and Mr. Fosbroke wrong, or the other way. It seems to me that if he be right the post of "officer of health" is a useless sinecure. Dr. Louis Parkes, in his work, "Hygiene and Public Health," says, "The mean duration of life or expectation of life at birth" is the same as mean age at death "when the population is stationary," that is, they coincide. He gives the mean duration of life in England (1871-80) for males as 41·35 years, and adds, "if the population were stationary, the mean age of males who died would have been 41·35, whereas the mean age at death was only 29 years." The village the water supply of which I had the honour to defend and my opponent the disgrace to traduce, shows some most instructive statistics. The parish register for 1873-82 has 48 deaths (including infants), numbering 2,109 years, an average in this stagnant village of nearly 44 years each death; but the years 1883-92 show 59 deaths and 3,344 years, that is, upwards of 55 years each death, or more than 13 years above the stagnant mean age, and 26 years above the floating mean. May I invite your judgment on this anomaly?

